# Needle and Branch Trait Variation Analysis and Associated SNP Loci Mining in *Larix olgensis*

**DOI:** 10.3390/ijms251810212

**Published:** 2024-09-23

**Authors:** Ying Cui, Jiawei Yan, Luping Jiang, Junhui Wang, Manman Huang, Xiyang Zhao, Shengqing Shi

**Affiliations:** 1 College of Forestry and Grassland, Jilin Agricultural University, Changchun 130118, China; cuiying@mails.jlau.edu.cn (Y.C.);; 2 State Key Laboratory of Tree Genetics and Breeding, Key Laboratory of Tree Breeding and Cultivation of State Forestry and Grassland Administration, Research Institute of Forestry, the Chinese Academy of Forestry, 1958 Box, Beijing 100091, China

**Keywords:** *Larix olgensis*, needle traits, branch traits, SNPs, GWAS, KASP markers

## Abstract

Needles play key roles in photosynthesis and branch growth in *Larix olgensis*. However, genetic variation and SNP marker mining associated with needle and branch-related traits have not been reported yet. In this study, we examined 131 samples of unrelated genotypes from *L. olgensis* provenance trails. We investigated phenotypic data for seven needle and one branch-related traits before whole genome resequencing (WGRS) was employed to perform a genome-wide association study (GWAS). Subsequently, the results were used to screen single nucleotide polymorphism (SNP) loci that were significantly correlated with the studied traits. We identified a total of 243,090,868 SNP loci, and among them, we discovered a total of 161 SNP loci that were significantly associated with these traits using a general linear model (GLM). Based on the GWAS results, Kompetitive Allele-Specific PCR (KASP), designed based on the DNA of population samples, were used to validate the loci associated with *L. olgensis* phenotypes. In total, 20 KASP markers were selected from the 161 SNPs loci, and BSBM01000635.1_4693780, BSBM01000114.1_5114757, and BSBM01000114.1_5128586 were successfully amplified, were polymorphic, and were associated with the phenotypic variation. These developed KASP markers could be used for the genetic improvement of needle and branch-related traits in *L. olgensis*.

## 1. Introduction

*Larix olgensis* A. Henry is a tall deciduous conifer tree species and the main native tree species used for afforestation and timber production in Northeast China, with a significant ornamental, economic, and ecological value [1]. Research on *L. olgensis* has mainly focused on conventional breeding, which is characterized by a long breeding cycle and low efficiency [2,3,4]. Molecular breeding techniques are modern biological breeding technology that uses molecular biology technology to carry out animal and plant breeding at the molecular level, including molecular marker-assisted breeding technology and genetic engineering breeding technology. We have done this by adding emphasis in line 35, marked in red. Advancements in molecular biology have led to the development of molecular breeding techniques, which offer powerful tools for enhancing forest genetic improvement. These techniques employ genetic markers such as randomly amplified polymorphc DNA (RAPD), restriction fragment length polymorphism (RFLP), simple sequence repeats (SSR), and single nucleotide polymorphism (SNP) markers. By incorporating these techniques, the accuracy of genetic analysis and the effectiveness of breeding can be significantly enhanced [5,6]. With the development of high-throughput sequencing technologies, SNP markers distributed across the entire genome have emerged as the third generation of molecular markers. They have been widely used in major candidate gene mapping, molecular marker-assisted selection (MAS) breeding, cultivar fingerprinting, etc. MAS, which is based on the close linkage between molecular markers and target traits, has emerged as a prominent approach in the study of quantitative traits [7,8]. At present, there are few studies on the genetic underlying larch needle and branch traits, and molecular markers associated with needle and branch traits have not been developed.

Leaves are the main organs where plant photosynthesis, respiration, and transpiration occur and the main site for energy synthesis and metabolism [9]. Diversity in leaf morphology can reflect genetic diversity, but it is also an important clue to understanding the genetic variation of plants [10]. However, previous studies on “larch” populations mainly focused on ecological traits and metabolomics [11,12], and only a few studies have implemented genetic analyses and molecular markers. Leaf photosynthetic pigments (such as chlorophylls and carotenoids) can absorb, transfer, and transform light energy and are an important index to measure plant photosynthesis and environmental stress [13,14,15]. Chlorophylls, fat-soluble plant pigments, comprise two major compounds: chlorophyll a (Chl a) and chlorophyll b (Chl b), which are green pigment photoreceptors present in all photosynthetic organisms [16]. Carotenoids comprise plant pigment compounds with a wide range of colors that act as accessory pigments to chlorophylls in photosynthesis [17]. Therefore, to understand the growth and physiological traits of *L. olgensis* needles, it is necessary to develop molecular markers and provide a scientific basis to enhance the genetic improvement of larch.

Genome-wide association studies (GWAS) have contributed to substantial advances in crop and forest tree research [7,18]. However, in conifers, whose chromosomes are 2*n* = 24, the contribution of GWAS has been more limited due to their large genome size (~10–40 Gb), which presents a significant challenge in developing a sufficient number of markers [19]. Furthermore, the number of trees genotyped is insufficient, with a typical sample size of less than 500 individuals [20,21]. In recent times, a number of reference genomes and transcriptome assemblies have become accessible for a number of tree species, such as *Picea abies* [22], *Pinus taeda* [23], *Picea glauca* [24], and *Pinus lambertiana* [25]. These recent advancements have enabled GWAS based on exome capture [26], genotyping-by-sequencing (GBS) [27], SNP arrays from transcripts [28], and resequencing [29]. However, high-throughput SNP genotyping is not the optimal approach when the number of target SNPs is in the hundreds or less due to its inappropriateness and lack of cost-effectiveness. A relatively low-cost genotyping approach, such as the Kompetitive Allele-Specific PCR (KASP) assay, is to be preferred in such cases. The KASP assay is a new genotyping method based on allele-specific amplification and high-sensitivity fluorescence detection. KASP-based genotyping is characterized by low cost, high throughput, and accurate double-allele genotyping of SNP and insertions-deletion (InDel) loci through specific matching of primer terminal bases. The method is widely used in the MAS selection of soybean, wheat, and other plants [30,31]. However, no study has been performed on the genome-wide identification of KASP markers with high polymorphism among resource materials or varieties that are capable of background screening in *L. olgensis*.

In this study, 131 *L. olgensis* germplasm resources were used as materials. We analyzed the variation of *L. olgensis* needle and branch traits and identified associated genetic polymorphisms through phenotypic and GWAS analyses. We developed corresponding KASP molecular markers corresponding to key SNP loci, and by detecting these loci, we can understand the phenotypic variation of plants and reduce the workload of breeding. This study provides a valuable reference for the genetic improvement of *L. olgensis*.

## 2. Results

### 2.1. Variation Analysis of Phenotypic Data

In this study, the variation in growth traits among 131 genotypes of *L. olgensis* was assessed (Table 1). The average needle length (NL) was 1.45 cm, with a range of 1.01 to 2.16 cm and a coefficient of variation (CV) of 15.17%. The average needle water content (NWC) was 55.19%, with a range of 38.28 to 71.21, and a CV of 8.73%. The average needle fascicles (NF) number was 9, with a range of 5 to 16, and a CV of 24.78%. The average biennial branch length (BBL) was 6.83 cm, with a range of 3.01 to 14.37, and a CV of 28.84%. The average Chl a relative content was 0.97%, with a range of 0.32 to 1.42, and a CV of 20.62%. The average Chl b relative content was 0.38%, with a range of 0.16 to 0.55, and a CV of 18.42%. The average Chl (a+b) relative content was 1.23%, with a range of 0.44 to 1.80, and a CV of 20.33%. The average Car relative content was 0.11%, with a range of 0.03 to 0.19, and a CV of 18.18%.

**Figure 1:**
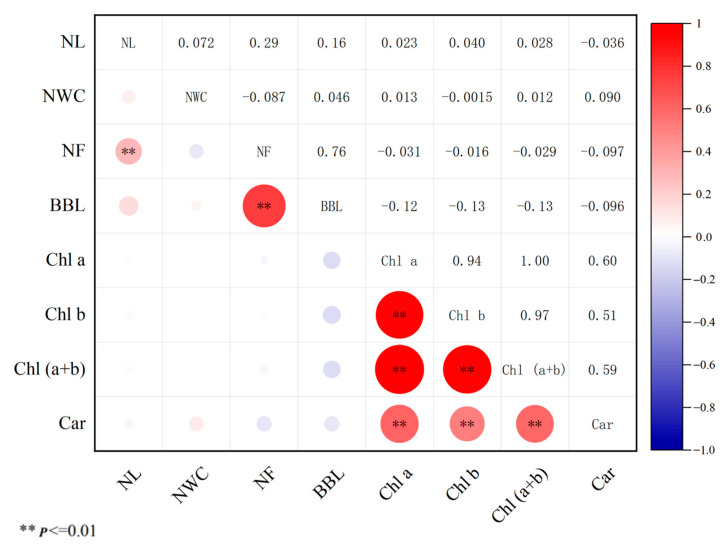
Phenotypic correlations among needle and branch traits. The blank squares indicate that there is no significant correlation (** *p* ≤ 0.01). NL: Needle length; NWC: Needle water content; NF: Needle fascicles; BBL: Biennial branch length; Chl a: Chlorophyll a; Chl b:Chlorophyll b; Chl (a+b): Chlorophyll (a+b); Car: Carotenoid.

**Figure 2:**
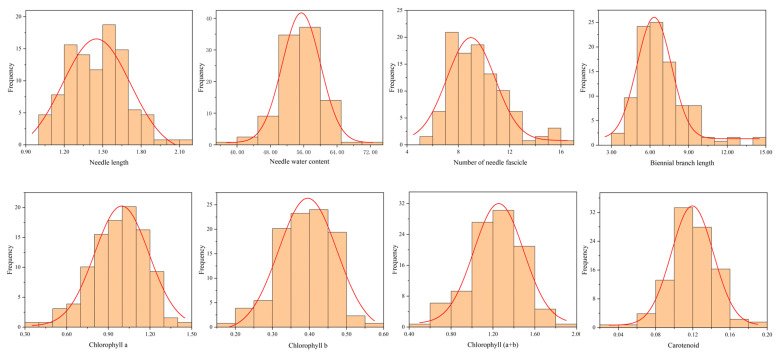
Frequency distribution of needle and branch traits in *Larix olgensis*.

Pearson’s correlation analysis was performed on needle and branch-related traits, and a cluster heat map of Pearson’s correlation coefficients among eight traits was constructed (Figure 1). In terms of needle and branch phenotypes, NF was positively correlated with the BBL (*p* < 0.01, *r* = 0.76), and NF was positively correlated with NL (*p* < 0.01, *r* = 0.29). Regarding photosynthetic pigment contents, there was a highly significant positive and significant correlation between Chl a, Chl b, Chl (a+b), and Car. The correlation coefficient between Chl a, Chl b, and Chl (a+b) was above 0.9. The Shapiro–Wilk test showed that the needle and branch traits also showed normal or near normal distributions (Figure 2), suitable for GWAS.

### 2.2. Analysis of Resequencing Data and SNP Screening

The resequencing raw data generated from the 131 genotypes was 116,699.59 Gb, and the filtered clean bases were 16,441.49 Gb, with an error rate of 0.02%. The proportion of Q20 bases was 98.69%, and the proportion of Q30 bases was 95.43%, indicating that the constructed library quality met the requirements for subsequent analysis of the resequenced samples. Ten thousand sequences were randomly selected from the fastq file of each sample, and blastn was used to compare the sequences to the NCBI NT database for contamination assessment. The results showed no significant contamination with sequences from other species in the sample sequences. Subsequently, the resequencing data from the 131 genotypes were compared with the reference genome of *L. olgensis*. The total sequencing data was 843,396,137 bp, the aligned sequences 841,468,984 bp, and the alignment rate was 99.77%. Moreover, the average sequencing depth of the samples was 9.28, and the coverage range was 85.29%, indicating that the sequencing data largely covered the reference genome and could be utilized for further analyses (Table 2).

A total of 1,157,702,025 SNPs were screened through rigorous filtering during the detection of SNP loci, then 243,090,868 SNPs were detected using VCFtool, with a genotype call rate ≥ 90%, MAF ≥ 0.05 and dimorphic SNP loci (detailed data not published, which was used for the 60K SNP array construction).

### 2.3. GWAS Analysis of Needle and Branch Traits

**Figure 3:**
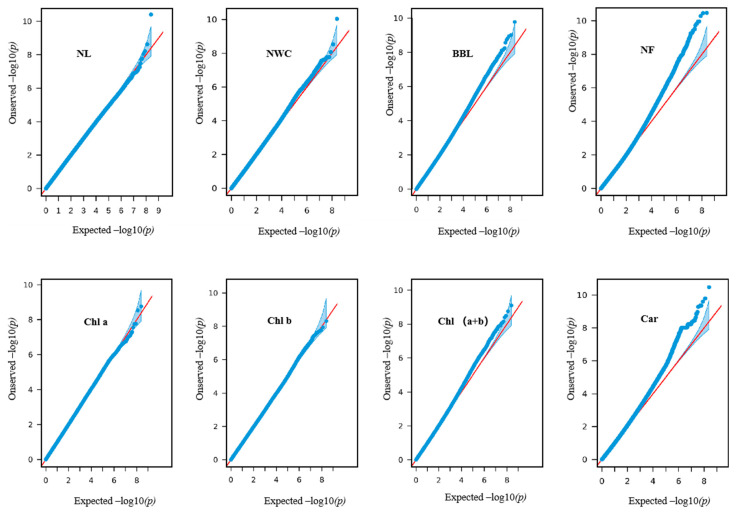
QQ plot results from the GWAS using the GLM model for needle and branch traits.

In this study, we integrated the phenotypic results for NL, NWC, NF, BBL, Chl a, Chl b, Chl (a+b), and Car with the WGRS data. We used a GLM to conduct GWAS employing the GAPIT package in R and created Manhattan plots and QQ plots representing the associated indicators (Supplementary Figure S1; Figure 3). A total of 161 SNPs were highly associated with seven needle and one branch-related traits in *L. olgensis* of which 153 SNP loci were associated with relative needle traits and eight SNP loci were associated with relative branch traits. SNPs with a significant association with needle and branch traits are detailed in Supplementary Table S1.

### 2.4. Validation Candidate SNPs by KASP Assay

**Figure 4:**
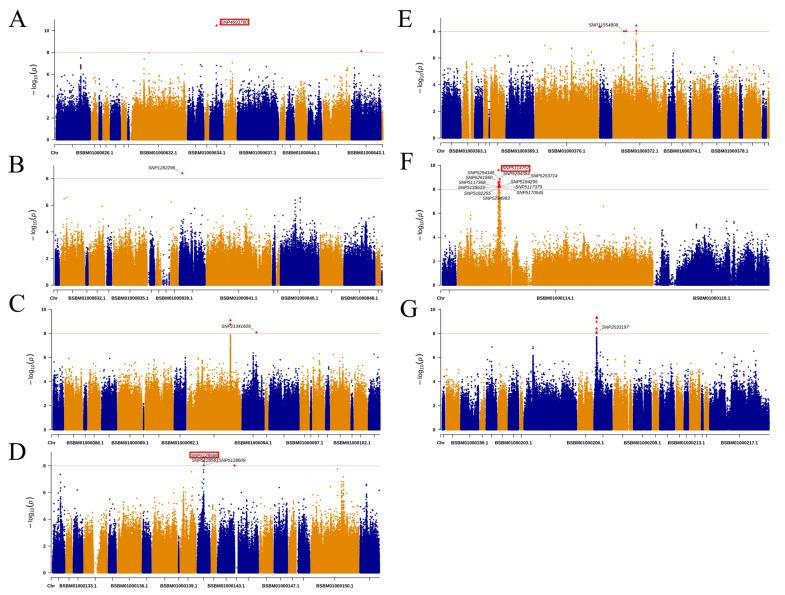
Manhattan map of KASP markers SNP loci. (**A**) Biennial branch length: SNP4693780. (**B**,**C**) Chlorophyll total: SNP1282296 and SNP1341609. (**D**–**G**) Carotenoid: SNP5128581, SNP 5128586, SNP5128609, SNP11554808, SNP5114757, SNP5294345, SNP5261660, SNP5117368, SNP5135633, SNP5182255, SNP5294993, SNP5117379, SNP5170545, and SNP5184295. The red frames are the KASP markers success loci.

**Figure 5:**
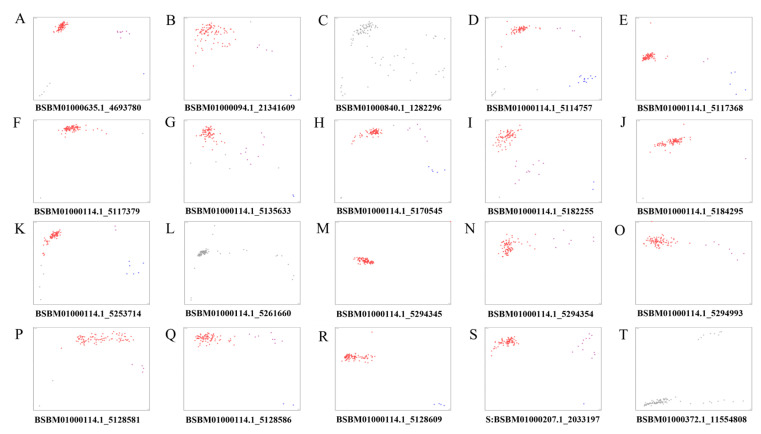
Genotyping of 20 KASP markers (**A**–**T**) The red, blue, purple, and black dots represent 5-Carboxyfluorescein (FAM), Hexachloro fluorescein (HEX), heterozygous, and unknown alleles, respectively.

Based on the results of the GWAS analysis, the 150 bp flanker sequence of the identified loci showing significant trait associations was extracted. The sequences of these loci were compared with the reference genome. Sequences with high copy numbers, high GC content, and many repeat sequences were removed (Figure 4), and 20 primers were designed and synthesized (Supplementary Table S2). 20 KASP markers linked to needle and branch traits were used for genotyping *L. olgensis*, and the genotyping results of the markers are shown in Figure 5. In the population samples, 11 markers were polymorphic, whereas 6 markers did not show any polymorphism.

### 2.5. Development of KASP Markers

**Figure 6:**
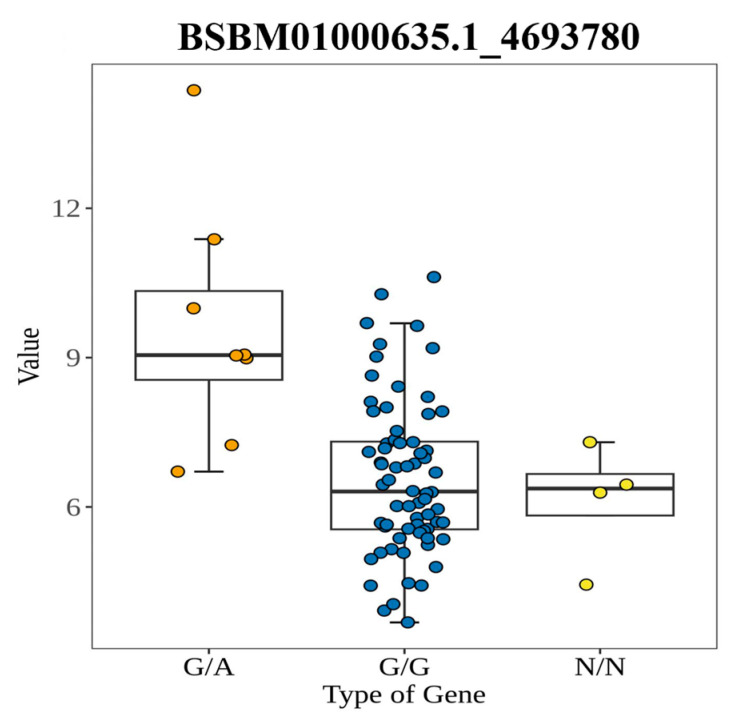
Box plot of biennial branch length labeled by KASP in *Larix. olgensis* population.

The polymorphic KASP primers were employed to assess associations of genetic variants with BBL in the population materials. Finally, an informative KASP marker BSBM01000635.1_4693780 was identified. The natural population, composed of 86 *L. olgensis* genotypes, was assessed, and a marker could be used to classify 82 *L. olgensis* genotypes, while the remaining 4 genotypes were not classified (N/N). BSBM01000635.1_4693780 differentiated 74 *L. olgensis* individuals with the genotype G/G and 8 with the genotype G/A (Figure 6).

**Figure 7:**
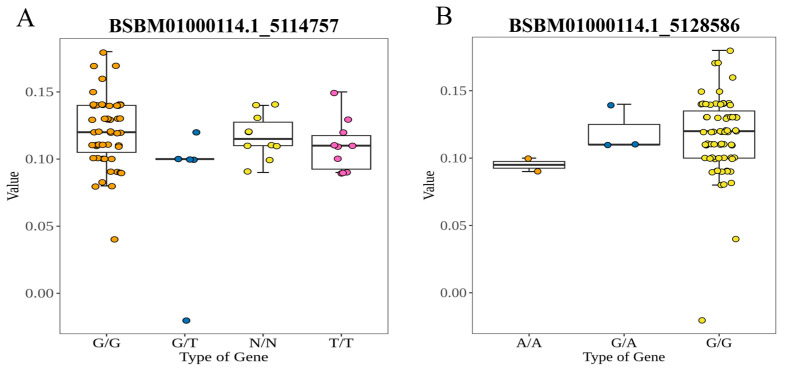
Box plot of carotenoid labeled by KASP in *Larix. olgensis* population. (**A**) Box plot of BSBM01000114.1_5114757 loci. (**B**) Box plot of BSBM01000114.1_5128586 loci.

The polymorphic KASP primers were also employed to assess genetic variants associated with Car content in the population Finally, 2 informative KASP markers BSBM01000114.1_5114757 and BSBM01000114.1_5128586, were identified. The natural population composed of 86 *L. olgensis* genotypes was assessed. The BSBM01000114.1_5114757 marker could be used to classify 75 *L. olgensis*, while the remaining 11 samples were not classified. It differentiated 58 *L. olgensis* individuals with the genotype GG, 5 with GT and 12 with TT (Figure 7A). The BSBM01000114.1_5128586 marker could be used to classify 86 *L. olgensis*. It differentiated 79 *L. olgensis* individuals with the genotype GG, 5 with genotype GA and 2 with the genotype AA (Figure 7B).

## 3. Discussion

Plant phenotypic variation is the result of the interaction between genetic diversity and the environment, and it also manifests in plant adaptation to the environment [32]. Studying plant phenotypic diversity can help understand the size of genetic variation in plant populations and also help in understanding the mode, mechanism, and influencing factors of plant adaptive evolution [33]. Perennial tree species have abundant phenotypic and genetic variation, which determines their adaptability to the environment and is the basis for maintaining the long-term stability of the forest ecosystem [34]. In this study, we determined the extent of genetic variation in 131 *L. olgensis* genotypes. Two traits varied greatly in the population, with a coefficient of variation greater than 20%. It is possible that the needle traits have been differentially influenced by complex climatic conditions, resulting in apparent differences in needle-related traits among the populations [35]. Chlorophyll plays a dominant role in photosynthesis, reflecting the plant’s ability to utilize and regulate light energy. In contrast, carotenoids play a secondary role as auxiliary pigments, which absorb visible light, after which light energy is transferred to chlorophyll, further improving the photosynthetic efficiency [36]. Plants with a greater chlorophyll content possess a more potent capacity to absorb light energy for the process of photosynthesis. The relatively high content of photosynthetic pigments in this study reflects the strong photosynthetic capacity of *L. olgensis* to a certain extent [37].

In the current study, we analyzed larch genotypes that are suitable for growing in northeast China. Such association studies have not previously explored the needle and branch-relates traits of *L. olgensis*. Therefore, our results may represent a promising and valuable resource of excellent loci associated with growth traits. However, the genomes of coniferous species are large and complex [22,23,24,25], with the *L. kaempferi* genome assembly at 10.97 Gb [38]. The development of molecular markers using traditional methods has a large and complex workload and is a lengthy process, while the number of markers obtained is not sufficient for the required sensitivity in association analyses [39]. Plant breeding techniques developed by leaders in the field are based on MAS approaches. Prior to MAS, the initial step involves the identification of DNA marker loci in the genome of forest trees, which are linked to specific wood traits. Variation in a limited number of genes can often lead to substantial phenotypic alterations [38]. SNP molecular markers are widely used for the construction of genetic maps, quantitative trait mapping analysis, and GWAS [40]. With the publication of the genomes of multiple species, the WGRS results can be compared with the existing reference genome sequences to identify genetic variations such as SNPs, InDels, and structural variants (SV) in the whole genome [21,41]. In coniferous trees, an SNP genotyping array developed by resequencing successfully genotyped 480 individuals [42]. Gulyaev et al. used more than 1 TB of WGRS data from 70 Salix taxa to identify SNPs on the autosomes and the chloroplast genomes for tree species phylogenetic analyses and to identify variants associated with different sex-determination systems in major groups of the genus [43].

A large number of SNP loci can be identified in a relatively small number of genotypes in conifers [44]. De la Torre et al. [45] identified 799 significant associations with cold tolerance-related traits by GWAS in 217 genotypes in Douglas-fir. The GoldenGate assay was used to genotype the offspring from three-generation outbred (G2) and inbred (F2). Based on 98 markers segregating in both pedigrees, a consensus map containing 357 SNPs from 292 different loci was generated [46]. In this study, the studied populations were mostly composed of individuals representing a species with a narrow distribution range. We selected 131 unrelated genotypes representing different natural forest populations that could represent the core germplasm of the northeastern part of China as determined by their limited genetic similarity. The results of the GWAS showed that SNP loci were significantly associated with abundant phenotypic variation. Thus, the results obtained demonstrate that sample size is not the most important factor in GWAS, and the genetic relationships among samples should be the focus of sample selection. Therefore, samples representing the core germplasm resources can be used as the materials for association analysis of the quantitative traits even when the availability of genetic materials is limited [47].

Currently, the assembly quality of the larch genome is inadequate, and the corresponding functional genes cannot be obtained [48]. KASP marker technology is of significant importance and has a wide range in various fields. It is extensively used for multiple purposes, including the identification of germplasm, the investigation of genetic relationships, the facilitation of breeding through molecular markers, the construction of genetic maps, and the mapping of genes [49,50,51]. KASP markers have been applied to locate candidate genes for yield traits such as plant height and thousand-grain weight [18]. In the current study, we developed three KASP markers BSBM01000635.1_4693780, BSBM01000114.1_5114757, and BSBM01000141.1_5128586, associated with BBL and Car. The development of KASP markers related to needle and branch traits of *L. olgensis* is of great practical significance for molecular marker breeding of *L. olgensis*.

## 4. Materials and Methods

### 4.1. Plant Materials

The population for the phenotype-genotype association analyses was derived from *L. olgensis* provenance trials established in Cuohai county, Heilongjiang province (122°51′ E, 47°27′ N) in 1980, which were originally distributed at 11 sites in the sites of Jilin and Heilongjiang provinces (Table 3). Seedlings were planted using a randomized complete block design with a density of 1 × 2 m [52]. In this study, representative individuals were selected based on the variation in needle and branch traits and whole genome resequencing (WGRS).

### 4.2. Phenotypic Traits Determination

In July 2023, which is the period of active growth, the traits of current-year needles and branches were not fully developed; thus, the traits of needles on biennial branches developed in the previous year were measured. The needle and biennial branch lengths were determined from each genotype using vernier calipers (each sample comprised 15 biological replicates) [53]. The number of needle fascicles on biennial branches was counted (each sample comprised 15 biological replicates). The fresh weight of the needles was weighed using an analytical balance. The measured needles were placed in a paper bag, sterilized at 105 °C for 15 min, and then dried at 85 °C in an oven to constant weight. The needles were placed in a dryer and cooled to room temperature. The dry weight of the needles was measured using an analytical balance, and the needle water content was calculated simultaneously (each sample comprised five biological replicates) [54]. Referring to the method of Cai et al., the needle water content was calculated as follows:

$$NWC=\frac{needle\;fresh\;weight-needle\;dry\;weight}{needle\;fresh\;weight}\times 100\%$$

The chlorophyll a (Chl a), chlorophyll b (Chl b), Chlorophyll total (Chl (a+b)), and carotenoid were determined by spectrophotometry (HD-UV90, China). The concentration of Chl a, Chl b, Chl (a+b), and Car were calculated according to Lichtenthaler [55] (Lichtenthaler, 1987):

Chl a = 13.95 × A665 − 6.08 × A649, Chl b = 24.69 × A649 − 7.32 × A665, Chl (a+b) = 7.05 × A6625 + 18.09 × A644.5, Car = (1000 × A470 − 2.05 × Chl a − 114.8 × Chl b)/245,

where A is the absorption at the corresponding wavelength; Chl a is the chlorophyll a concentration; Chl b is the chlorophyll b concentration; Chl (a+b) is the total chlorophyll concentration; Car is the total carotenoid concentration. Pigment concentrations were expressed as μg mL^−1^ of diluted extract.

### 4.3. DNA Extraction

In July 2023, the genomic DNA was extracted from *L. olgensis* needles using the magnetic beads method [56]. The DNA quality and concentration of DNA were assessed using 1.0% agarose gel electrophoresis and an ND-1000 spectrophotometer, respectively. After the extraction quality was determined, the DNA solution was diluted to 20–100 ng/μL used as the working solution and stored at −20°C for subsequent detection analyses.

### 4.4. WGRS and SNP Calling

The DNAs of 131 *L. olgensis* genotypes were resequenced using the DNBSEQ-T7 platform (MGI, Shenzhen, China) with an expected target coverage of 10× in Huazhi Bio-Tech (Changsha, China). The raw data were filtered using the fastp software (v2.20.0) (parameter Q30, the rest were the default) to obtain clean read data [57]. The clean reads were then aligned to the *L. kaempferi* reference genome (GCA_027924585.1) using the Burrows-Wheeler Aligner (BWA) software (version BWA-0.7.17(r1188)) [38]. SNP-calling was performed using the genome analysis toolkit software (GATK4.3.0.0) [58]. Finally, raw SNPs were filtered using the VCFtools software (v0.1.15) [59] based on the following criteria: deletion call rate ≥ 0.9, minor allele frequency (MAF) ≥ 0.05, loci with only two alleles were retained, and information in all INFO field were retained without filtering. The high-quality SNPs obtained through these filtering criteria were used for GWAS analysis.

### 4.5. Genome-Wide Association Analysis

GWAS was performed using the R package rMVP (version 1.1.1), which employs a general linear model (GLM) [60]. The SNP loci significantly associated with the target traits were determined based on −Log*p* values ≥ 8 as the threshold. The QQ map and the Manhattan map were drawn using the CMPlot (version 4.5.1) of the R package. The Manhattan plot was plotted using rMVp to visualize the GWAS analysis.

### 4.6. KASP Genotyping Assay

Using the Primer-BLAST function of NCBI (https://www.ncbi.nlm.nih.gov/, accessed on 19 April 2024), KASP-PCR amplification primers were designed based on the SNP loci. Twenty of the designed primers were significantly associated with *L. olgensis* BBL, Chl (a+b), and Car. Each pair of primers consisted of two specific forward primers F1 and F2, and a generic reverse primer, R F1 and F2, which contained 6-carboxyfluorescein (FAM) and hexachloro-6-methylfluorescein (HEX) fluorescent linker sequences (underlined), respectively.

KASP labeling validation was performed on 96 samples in Douglas Scientific’s Array Tape system. The temperature cycling conditions were predenaturation at 94 °C for 15 min, followed by 30 cycles of denaturation at 95 °C for 20 s, extension at 65–56 °C for 1 min, with a decrease of 0.8°C per cycle for 10 cycles, denaturation at 94 °C for 20 s, and extension at 57 °C for 1 min.

### 4.7. Statistical Analysis of Needle Traits

IBM SPSS Statistics 26 software was used to calculate the mean value, standard deviation, and coefficient of variation (CV) of each measured trait. The Origin 2021 software was used to determine the frequency distribution and correlation analysis and to generate the figures.

## 5. Conclusions

This study identified 161 SNP loci associated with the seven-needle and one-branch-related traits in *L. olgensis*. Genotypic and phenotypic variability was combined to conduct GWAS, and KASP markers were developed based on the significantly associated loci. These significant KASP markers could be used to genotype *L. olgensis* individuals accurately. In conclusion, this work has enriched the phenotypic and genotypic data of *L. olgensis* and provided valuable information for further studies on the regulation of needle and branch traits in *L. olgensis*. Additionally, further research is needed to explore these loci and gain a deeper understanding of the underlying molecular mechanisms in regulating the needle and branch traits of *L. olgensis*. These findings contribute to a better understanding of the regulatory mechanisms of coniferous tree needle and branch-related traits, offering a scientific basis for optimizing larch’s growth traits. This study provides valuable genetic resources and a solid theoretical basis for molecular design breeding and marker-assisted breeding of *L. olgensis*. It can improve the yield of *L. olgensis* with excellent wood properties, reduce the workload of breeders, and accelerate the breeding process of trees.

## Figures and Tables

**Table 1:** 131 *Larix olgensis* resources of needle and branch traits basic statistics.

Trait	Mean	SD	Range	CV (%)	Kurtosis	Skewness
Needle length (cm)	1.45	0.22	1.01~2.16	15.17	0.06	0.35
Needle water content (%)	55.19	4.82	38.28~71.21	8.73	1.58	−0.15
Number of needle fascicles	9	2.23	5~16	24.78	0.74	0.88
Biennial branch length (cm)	6.83	1.97	3.01~14.37	28.84	2.69	1.32
Chlorophyll a (%)	0.97	0.20	0.32~1.42	20.62	0.22	−0.46
Chlorophyll b (%)	0.38	0.07	0.16~0.55	18.42	1.80	0.19
Chlorophyll (a+b) (%)	1.23	0.25	0.44~1.80	20.33	0.19	−0.43
Carotenoid (%)	0.11	0.02	0.03~0.19	18.18	1.14	−0.06

**Table 2:** Resequencing data statistics.

Whole Genome Resequencing		Comparison of Reference Genome		Variant Detection	
Raw base (Gb)	16,699.59	Total bases (bp)	16,441.49	Homozygosis (RR) (Gb)	19.95
Clean base (Gb)	16,441.49	Mapped bases (bp)	16,403.70	Homozygosis (AA) (Gb)	3.96
Clean rate (%)	98.46	Mapping rate (%)	99.77	Heterozygosis (RA) (Gb)	7.28
Q20 base rate (%)	98.69	Average depth (X)	9.28	Missing (Gb)	0.66
Q30 base rate (%)	95.44	Coverage at least 1X (%)	85.29	Ratio (%)	97.92

**Table 3:** Geographical locations of provenances and collection quantity.

Provenance	Abbreviation	Longitude (E)	Latitude (N)	Altitude (m)	Collection Quantity
BaiDaoShan	BDS	131°07′	44°00′	702	12
BaiHe	BH	128°08′	42°25′	719	9
DaHaiLin	DHL	129°48′	44°28′	570	14
DaShiTou	DST	128°31′	43°19′	950	14
HeLong	HL	129°01′	42°30′	542	14
JiXi	JX	130°50′	45°12′	326	13
LuShuiHe	LSH	127°48′	42°30′	762	12
MuLing	ML	130°31′	44°52′	327	12
TianQiaoLing	TQL	129°17′	43°34′	967	11
XiaoBeiHu	XBH	128°50′	44°01′	827	12
LongJiang	LG	122°51′	47°27′	340	8

## Data Availability

Data are contained within the article and Supplementary Materials.

## Supplementary Materials

The following supporting information can be downloaded at: https://www.mdpi.com/article/10.3390/ijms251810212/s1.
