# Supplementary material for: Needle and Branch Trait Variation Analysis and Associated SNP Loci Mining in Larix olgensis
Source: Int J Mol Sci. 2024 Sep 23;25(18):10212. doi: 10.3390/ijms251810212 (PMC11432355; doi:10.3390/ijms251810212)
Supplement: Supplementary file 1 [file ijms-25-10212-s001.zip › Figure S1.pdf]

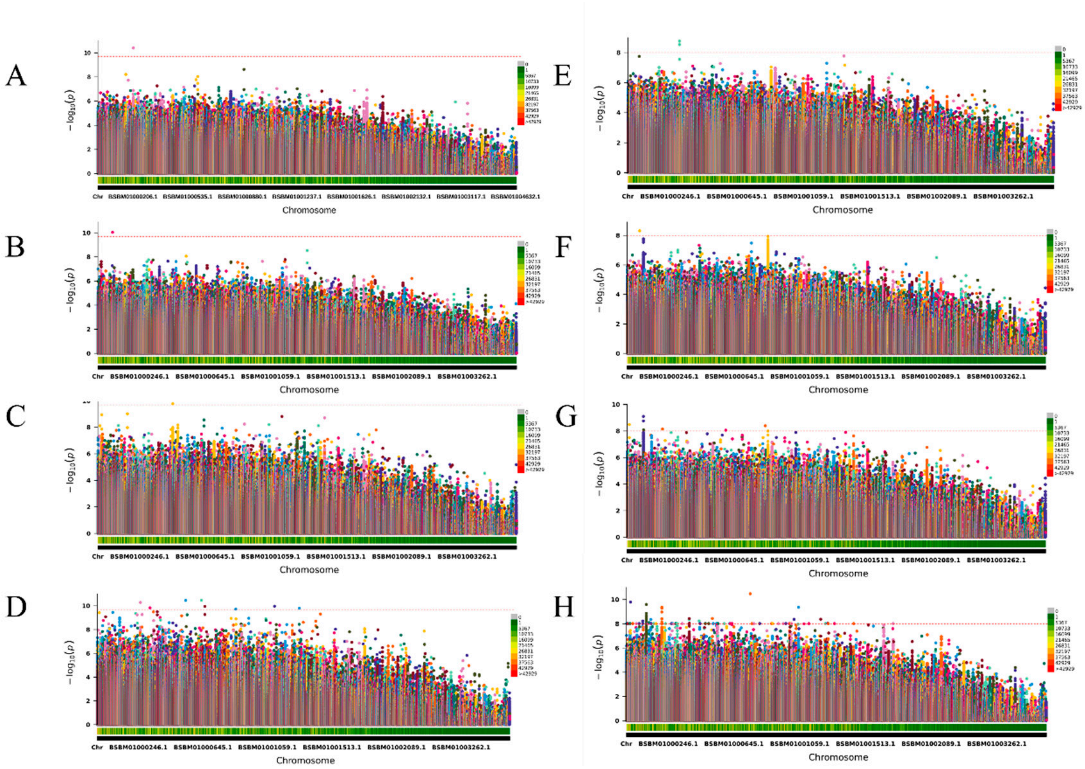

Figure S1. GWAS results of needle and branch traits in *Larix olgensis*. (A-H) respectively is needle length, needle water content, needle fascicle, biennial branch length, chlorophyll a, chlorophyll b, chlorophyll total, carotenoid.
