# Supplementary material for: Needle and Branch Trait Variation Analysis and Associated SNP Loci Mining in Larix olgensis
Source: Int J Mol Sci. 2024 Sep 23;25(18):10212. doi: 10.3390/ijms251810212 (PMC11432355; doi:10.3390/ijms251810212)
Supplement: Supplementary file 1 [file ijms-25-10212-s001.zip › Table S1.pdf]

Table S1. Single nucleotide polymorphisms with significant needle and branch traits associations in 131 *Larix olgensis* genotypes based on GWAS

Trait	Chr.	Position	Allele	P-value
Needle length	BSBM01000208.1	2980219	G/A	3.97E-11
Needle water content	BSBM01000085.1	5103979	C/A	9.22E-11
Needle fascicles	BSBM01000445.1	29619081	A/T	1.70E-10
Biennial branch length	BSBM01000258.1	4004035	G/A	5.25E-11
Biennial branch length	BSBM01000316.1	15155592	C/T	1.48E-10
Biennial branch length	BSBM01000535.1	7422662	G/T	3.52E-11
Biennial branch length	BSBM01000635.1	4693780	G/A	3.41E-11
Biennial branch length	BSBM01000658.1	1420386	C/A	1.13E-10
Biennial branch length	BSBM01000857.1	2804765	G/C	1.92E-10
Biennial branch length	BSBM01001108.1	460345	G/T	1.09E-10
Biennial branch length	BSBM01001280.1	2326935	G/A	1.60E-10
Chlorophyll A	BSBM01000299.1	33051047	T/C	1.72E-09
Chlorophyll A	BSBM01000299.1	33051055	G/A	2.93E-09
Chlorophyll B	BSBM01000072.1	17750955	G/T	4.85E-09
Chlorophyll total	BSBM01000012.1	3915658	G/A	3.25E-09
Chlorophyll total	BSBM01000093.1	64371138	G/A	7.97E-10
Chlorophyll total	BSBM01000093.1	64372554	G/T	1.76E-09
Chlorophyll total	BSBM01000094.1	21341609	G/A	8.24E-09
Chlorophyll total	BSBM01000207.1	2072122	G/A	7.06E-09
Chlorophyll total	BSBM01000591.1	2165379	G/A	8.70E-09
Chlorophyll total	BSBM01000840.1	1282296	G/A	4.05E-09
Carotenoid	BSBM01000001.1	77919964	A/C	9.60E-09
Carotenoid	BSBM01000009.1	2681096	T/C	9.60E-09
Carotenoid	BSBM01000009.1	2681518	A/C	9.60E-09
Carotenoid	BSBM01000009.1	2690200	C/T	9.60E-09
Carotenoid	BSBM01000020.1	6427382	C/A	9.60E-09
Carotenoid	BSBM01000023.1	1423966	A/G	1.61E-10
Carotenoid	BSBM01000036.1	16923894	T/A	9.60E-09
Carotenoid	BSBM01000057.1	16923894	C/T	9.60E-09
Carotenoid	BSBM01000073.1	24013493	G/T	9.60E-09
Carotenoid	BSBM01000093.1	34326599	C/T	9.60E-09
Carotenoid	BSBM01000093.1	65340770	G/A	5.98389E-09
Carotenoid	BSBM01000114.1	5084156	G/A	6.1326E-09
Carotenoid	BSBM01000114.1	5105982	C/T	6.1326E-09
Carotenoid	BSBM01000114.1	5108876	G/T	6.1326E-09
Carotenoid	BSBM01000114.1	5108989	C/T	6.1326E-09
Carotenoid	BSBM01000114.1	5114679	G/A	2.74377E-09
Carotenoid	BSBM01000114.1	5114757	G/T	2.53603E-10
Carotenoid	BSBM01000114.1	5117338	G/A	6.1326E-09
Carotenoid	BSBM01000114.1	5117368	C/T	6.1326E-09
Carotenoid	BSBM01000114.1	5117379	C/A	6.1326E-09
Carotenoid	BSBM01000114.1	5135633	G/A	6.1326E-09
Carotenoid	BSBM01000114.1	5170055	T/A	6.1326E-09
Carotenoid	BSBM01000114.1	5170545	G/T	6.1326E-09
Carotenoid	BSBM01000114.1	5171968	C/G	6.1326E-09
Carotenoid	BSBM01000114.1	5175658	G/A	6.1326E-09
Carotenoid	BSBM01000114.1	5180782	G/A	7.23097E-09
Carotenoid	BSBM01000114.1	5180928	G/T	6.1326E-09
Carotenoid	BSBM01000114.1	5182255	G/A	6.1326E-09

Carotenoid	BSBM01000114.1	5183980	G/A	3.60274E-09
Carotenoid	BSBM01000114.1	5184295	C/T	5.9845E-09
Carotenoid	BSBM01000114.1	5238815	C/T	4.12188E-09
Carotenoid	BSBM01000114.1	5238926	C/T	6.78806E-09
Carotenoid	BSBM01000114.1	5239003	C/T	6.78806E-09
Carotenoid	BSBM01000114.1	5248656	G/T	4.78915E-09
Carotenoid	BSBM01000114.1	5253645	C/T	6.1326E-09
Carotenoid	BSBM01000114.1	5253714	C/A	6.1326E-09
Carotenoid	BSBM01000114.1	5261660	C/G	2.26434E-09
Carotenoid	BSBM01000114.1	5294345	T/C	1.37255E-09
Carotenoid	BSBM01000114.1	5294354	C/G	1.37255E-09
Carotenoid	BSBM01000114.1	5294993	G/A	6.1326E-09
Carotenoid	BSBM01000114.1	5295836	C/T	6.1326E-09
Carotenoid	BSBM01000123.1	10478652	C/G	9.59595E-09
Carotenoid	BSBM01000123.1	10478674	A/C	9.59595E-09
Carotenoid	BSBM01000123.1	10478678	A/T	9.59595E-09
Carotenoid	BSBM01000123.1	10482094	C/T	9.59595E-09
Carotenoid	BSBM01000125.1	9128477	G/A	9.59595E-09
Carotenoid	BSBM01000125.1	9128478	C/A	9.59595E-09
Carotenoid	BSBM01000125.1	9128681	A/G	9.59595E-09
Carotenoid	BSBM01000125.1	9128682	C/G	9.59595E-09
Carotenoid	BSBM01000127.1	19435663	T/C	9.59595E-09
Carotenoid	BSBM01000130.1	12375077	G/T	8.05557E-09
Carotenoid	BSBM01000141.1	5116427	A/G	4.43538E-09
Carotenoid	BSBM01000141.1	5128581	T/C	9.39186E-09
Carotenoid	BSBM01000141.1	5128586	A/G	9.39186E-09
Carotenoid	BSBM01000141.1	5128609	G/C	9.39186E-09
Carotenoid	BSBM01000143.1	13356382	A/G	9.59595E-09
Carotenoid	BSBM01000158.1	18946839	C/G	9.59595E-09
Carotenoid	BSBM01000173.1	13189005	A/C	9.59595E-09
Carotenoid	BSBM01000207.1	2014430	C/A	1.07E-09
Carotenoid	BSBM01000207.1	2028551	A/C	3.84795E-09
Carotenoid	BSBM01000207.1	2033197	G/T	9.76416E-09
Carotenoid	BSBM01000207.1	2040502	G/A	7.50259E-09
Carotenoid	BSBM01000207.1	2073361	T/G	5.22003E-10
Carotenoid	BSBM01000207.1	2079949	C/T	3.80098E-09
Carotenoid	BSBM01000207.1	2091118	G/A	9.76416E-09
Carotenoid	BSBM01000207.1	2114845	G/A	4.36145E-10
Carotenoid	BSBM01000207.1	2175886	G/A	4.97959E-10
Carotenoid	BSBM01000207.1	2206049	A/C	4.02593E-09
Carotenoid	BSBM01000229.1	3282403	T/G	9.59595E-09
Carotenoid	BSBM01000264.1	21924448	C/G	9.59595E-09
Carotenoid	BSBM01000264.1	21924481	C/T	9.59595E-09
Carotenoid	BSBM01000272.1	8992585	G/A	9.59595E-09
Carotenoid	BSBM01000272.1	9018216	A/T	9.59595E-09
Carotenoid	BSBM01000289.1	22965296	A/G	9.59595E-09
Carotenoid	BSBM01000291.1	17950990	C/T	9.59595E-09
Carotenoid	BSBM01000299.1	52217903	C/T	9.59595E-09

Carotenoid	BSBM01000328.1	7935344	G/C	9.59595E-09
Carotenoid	BSBM01000371.1	226138	C/T	4.54974E-09
Carotenoid	BSBM01000372.1	11554808	C/T	9.74729E-09
Carotenoid	BSBM01000372.1	13928738	C/A	9.59595E-09
Carotenoid	BSBM01000372.1	13928745	G/T	9.59595E-09
Carotenoid	BSBM01000372.1	24687478	G/A	3.70838E-09
Carotenoid	BSBM01000372.1	24687963	A/G	9.11401E-09
Carotenoid	BSBM01000431.1	10230595	G/C	5.89305E-09
Carotenoid	BSBM01000436.1	17454653	G/A	9.59595E-09
Carotenoid	BSBM01000467.1	4280805	T/A	9.59595E-09
Carotenoid	BSBM01000487.1	9487061	G/C	9.59595E-09
Carotenoid	BSBM01000510.1	16088421	C/A	3.66108E-09
Carotenoid	BSBM01000515.1	4519400	A/G	9.59595E-09
Carotenoid	BSBM01000535.1	7818358	T/G	9.59595E-09
Carotenoid	BSBM01000545.1	2505379	A/G	9.59595E-09
Carotenoid	BSBM01000545.1	2505394	A/T	9.59595E-09
Carotenoid	BSBM01000545.1	2505396	G/A	9.59595E-09
Carotenoid	BSBM01000545.1	2505415	A/G	9.59595E-09
Carotenoid	BSBM01000545.1	2505512	A/G	9.59595E-09
Carotenoid	BSBM01000545.1	2505521	C/T	9.59595E-09
Carotenoid	BSBM01000545.1	2505553	T/G	9.59595E-09
Carotenoid	BSBM01000584.1	3881782	T/G	9.59595E-09
Carotenoid	BSBM01000584.1	3881791	G/A	9.59595E-09
Carotenoid	BSBM01000584.1	3881792	T/C	9.59595E-09
Carotenoid	BSBM01000618.1	3691363	C/A	9.59595E-09
Carotenoid	BSBM01000618.1	3691383	G/A	9.59595E-09
Carotenoid	BSBM01000622.1	23856713	A/G	9.59595E-09
Carotenoid	BSBM01000632.1	23592298	T/C	9.59595E-09
Carotenoid	BSBM01000748.1	1243095	A/C	3.32271E-11
Carotenoid	BSBM01000758.1	1770534	T/C	9.59595E-09
Carotenoid	BSBM01000769.1	15325888	T/C	9.59595E-09
Carotenoid	BSBM01000769.1	15325924	T/G	9.59595E-09
Carotenoid	BSBM01000805.1	20032337	G/A	9.59595E-09
Carotenoid	BSBM01000805.1	20049371	A/C	9.59595E-09
Carotenoid	BSBM01000806.1	8602755	C/T	9.59595E-09
Carotenoid	BSBM01000806.1	8627811	T/C	9.59595E-09
Carotenoid	BSBM01000806.1	8630270	C/A	9.59595E-09
Carotenoid	BSBM01000806.1	8635830	C/G	9.59595E-09
Carotenoid	BSBM01000882.1	2762496	C/T	9.59595E-09
Carotenoid	BSBM01000888.1	529355	G/A	9.59595E-09
Carotenoid	BSBM01000924.1	7016335	A/G	9.59595E-09
Carotenoid	BSBM01000924.1	7016336	T/C	9.59595E-09
Carotenoid	BSBM01000955.1	3516848	A/T	9.59595E-09
Carotenoid	BSBM01000955.1	3516849	A/G	9.59595E-09
Carotenoid	BSBM01000971.1	632668	A/G	9.59595E-09
Carotenoid	BSBM01000971.1	632672	A/C	9.59595E-09
Carotenoid	BSBM01000971.1	632696	G/T	9.59595E-09
Carotenoid	BSBM01000971.1	672738	G/C	9.59595E-09

Carotenoid	BSBM01000993.1	3054361	G/C	9.59595E-09
Carotenoid	BSBM01000993.1	3105779	C/A	9.59595E-09
Carotenoid	BSBM01000993.1	3235873	T/C	9.59595E-09
Carotenoid	BSBM01000993.1	6309415	G/A	9.59595E-09
Carotenoid	BSBM01001000.1	19356817	A/G	9.59595E-09
Carotenoid	BSBM01001005.1	40920656	T/C	9.59595E-09
Carotenoid	BSBM01001023.1	9938566	A/G	4.26829E-09
Carotenoid	BSBM01001051.1	7025137	C/A	4.37405E-10
Carotenoid	BSBM01001121.1	2261843	C/T	9.59595E-09
Carotenoid	BSBM01001199.1	3200945	C/T	4.24576E-09
Carotenoid	BSBM01001231.1	283721	C/A	9.59595E-09
Carotenoid	BSBM01001248.1	2026076	G/A	8.92891E-09
Carotenoid	BSBM01001252.1	3176369	C/T	9.59595E-09
Carotenoid	BSBM01001296.1	3238252	T/C	9.59595E-09
Carotenoid	BSBM01001430.1	10143489	A/G	9.59595E-09
Carotenoid	BSBM01001719.1	15300754	G/A	9.59595E-09
