# Supplementary material for: Needle and Branch Trait Variation Analysis and Associated SNP Loci Mining in Larix olgensis
Source: Int J Mol Sci. 2024 Sep 23;25(18):10212. doi: 10.3390/ijms251810212 (PMC11432355; doi:10.3390/ijms251810212)
Supplement: Supplementary file 1 [file ijms-25-10212-s001.zip › Table S2.pdf]

Table S2. 20 KASP primer sequences

Trait	Prime name	Prime sequence	Allele
Biennial	BSBM01000635.1_4693780	F1: **GAAGGTGACCAAGTTCATGCT**ATGCGCCAATTTCTTGAAGGATAA**G**	G
branch		F2: **GAAGGTCGGAGTCAACGGATT**ATGCGCCAATTTCTTGAAGGATAA**A**	A
length		R: TGCCAAATTACTCAAACGCCACTGTA	
Chlorophyll	BSBM01000094.1_1341609	F1: **GAAGGTGACCAAGTTCATGCT**GCCAATGATCAGAGCCAGCA**G**	G
total		F2: **GAAGGTCGGAGTCAACGGATT**GCCAATGATCAGAGCCAGCA**A**	A
		R: TTGCTTGTGATAGGGGTGAAGTCAAC	
Chlorophyll	BSBM01000840.1_1282296	F1: **GAAGGTGACCAAGTTCATGCT**CAATTCAGTCCCCAACTAAAAGAAGTACT**G**	G
total		F2: **GAAGGTCGGAGTCAACGGATT**GCAATTCAGTCCCCAACTAAAAGAAGTACT**A**	A
		R: TGGTAGACAAAGCATTCTGGCTCACT	
Carotenoid	BSBM01000114.1_5114757	F1: **GAAGGTGACCAAGTTCATGCT**CGATGCAAAACGCAACTATGAA**G**	G
		F2: **GAAGGTCGGAGTCAACGGATT**GCGATGCAAAACGCAACTATGAA**T**	T
		R: AACTTTTGAGACTTTTCGCGCGATT	
Carotenoid	BSBM01000114.1_5117368	F1: **GAAGGTGACCAAGTTCATGCT**GGGCCCCGTGTTCCAAT**C**	C
		F2: **GAAGGTCGGAGTCAACGGATT**GGGCCCCGTGTTCCAAT**T**	T
		R: TGTCTTGTTCCTAGGGTTTGGATGAGA	
Carotenoid	BSBM01000114.1_5117379	F1: **GAAGGTGACCAAGTTCATGCT**CCGTGTTCCAATCTCCAATCAGT**C**	C
		F2: **GAAGGTCGGAGTCAACGGATT**CCGTGTTCCAATCTCCAATCAGT**A**	A
		R: TGTCTTGTTCCTAGGGTTTGGATGAGA	
Carotenoid	BSBM01000114.1_5135633	F1: **GAAGGTGACCAAGTTCATGCT**TCCTTGCACATTCTAAACTGCTCAT**C**	C
		F2: **GAAGGTCGGAGTCAACGGATT**TCCTTGCACATTCTAAACTGCTCAT**T**	T
		R: ATCGGTTTCTGACTTCATGGATGGAT	
Carotenoid	BSBM01000114.1_5170545	F1: **GAAGGTGACCAAGTTCATGCT**CTTTAGAAAGACTCTTCGACTTCAACAATT**G**	G
		F2: **GAAGGTCGGAGTCAACGGATT**ACTTTAGAAAGACTCTTCGACTTCAACAATT**T**	T
		R: GGTCTGCATTGGGAAACGTCTAGATC	
Carotenoid	BSBM01000114.1_5182255	F1: **GAAGGTGACCAAGTTCATGCT**AACTTCGTACTCCATCAATGTCAAC**G**	G
		F2: **GAAGGTCGGAGTCAACGGATT**CATAACTTCGTACTCCATCAATGTCAAC**A**	A
		R: ATCGTTATAGCATTGGGGAGGCTAGC	
Carotenoid	BSBM01000114.1_5184295	F1: **GAAGGTGACCAAGTTCATGCT**TCTGATCACTCAGGTTACATGGACTTT**C**	C
		F2: **GAAGGTCGGAGTCAACGGATT**TTCTGATCACTCAGGTTACATGGACTTT**T**	T
		R: GAACCAACAAGAGGAATCCTTGAGGA	
Carotenoid	BSBM01000114.1_5253714	F1: **GAAGGTGACCAAGTTCATGCT**GTCGGAAACACTCCAGCATCAA**C**	C
		F2: **GAAGGTCGGAGTCAACGGATT**TCGGAAACACTCCAGCATCAA**A**	A
		R: ATGGGTTGATTGGTGATCATCTTTCC	
Carotenoid	BSBM01000114.1_5261660	F1: **GAAGGTGACCAAGTTCATGCT**ATTTGGAAATGCCCACCAAGA**G**	G
		F2: **GAAGGTCGGAGTCAACGGATT**ATTTGGAAATGCCCACCAAGA**C**	C
		R: TGGAAACCTTAACAAAGAATGGATCCA	
Carotenoid	BSBM01000114.1_5294345	F1: **GAAGGTGACCAAGTTCATGCT**ATACTCCACATATGGTGCGGGAG**T**	T
		F2: **GAAGGTCGGAGTCAACGGATT**CCACATATGGTGCGGGAG**C**	C
		R: AAACCACGCAACAAATTACTGTGCAC	
Carotenoid	BSBM01000114.1_5294354	F1: **GAAGGTGACCAAGTTCATGCT**TGCGGGAGTGGTTGGTG**C**	C
		F2: **GAAGGTCGGAGTCAACGGATT**TGCGGGAGTGGTTGGTG**G**	G
		R: CGCTCCTAAACCACGCAACAAATTAC	
Carotenoid	BSBM01000114.1_5294993	F1: **GAAGGTGACCAAGTTCATGCT**AAAGTAGAGGTCATGGGAGTCTCC**G**	G
		F2: **GAAGGTCGGAGTCAACGGATT**AAAAGTAGAGGTCATGGGAGTCTCC**A**	A
		R: ACCCACATATTTGGTTTCCAACGTTG	
Carotenoid	BSBM01000141.1_5128581	F1: **GAAGGTGACCAAGTTCATGCT**GTGCTCACCAGCCCTCACTG**T**	T
		F2: **GAAGGTCGGAGTCAACGGATT**GCTCACCAGCCCTCACTG**C**	C
		R: GTCGCCTTCTTCGGTAGGGTGAC	
Carotenoid	BSBM01000141.1_5128586	F1: **GAAGGTGACCAAGTTCATGCT**CCAGCCCTCACTGTTGCC**A**	A
		F2: **GAAGGTCGGAGTCAACGGATT**CAGCCCTCACTGTTGCC**G**	G

	R: GTCGCCTTCTTCGGTAGGGTGAC	
Carotenoid BSBM01000141.1_5128609	F1: **GAAGGTGACCAAGTTCATGCT**CCGTCACCCTACCGAAGAAGGC**G**	G
	F2: **GAAGGTCGGAGTCAACGGATT**GTCACCCTACCGAAGAAGGC**C**	C
	R: AGATCTCGGTGTTGCAGCTCCAG	
Carotenoid BSBM01000207.1_2033197	F1: **GAAGGTGACCAAGTTCATGCT**GTTATCCTATCGAGGATGGTAGAGGTGTA**G**	G
	F2: **GAAGGTCGGAGTCAACGGATT**GTTATCCTATCGAGGATGGTAGAGGTGTA**T**	T
	R: TCTCTTGGGGACCCACCTCTTTAGTT	
Carotenoid BSBM01000372.1_11554808	F1: **GAAGGTGACCAAGTTCATGCT**AGTGATATGACACAAATGCCCAAAGT**T**	T
	F2: **GAAGGTCGGAGTCAACGGATT**GTGATATGACACAAATGCCCAAAGT**C**	C
	R: ACACATCCTATGCCGTGATGTTGTTC	
